# Evidence-based circumcision policy for Australia

**DOI:** 10.31083/j.jomh1806132

**Published:** 2022-05-30

**Authors:** Brian J. Morris, Athos Katelaris, Norman J. Blumenthal, Mohamed Hajoona, Adrian C. Sheen, Leslie Schrieber, Eugenie R. Lumbers, Alex D. Wodak, Phillip Katelaris

**Affiliations:** 1 School of Medical Sciences, University of Sydney, Sydney, NSW 2006, Australia; 2 Department of Urology, St George Hospital, Sydney, NSW 2217, Australia; 3 Department of Obstetrics and Gynaecology, SAN Clinic, Wahroonga, NSW 2076, Australia; 4 Victoria Circumcision Clinic, The Regent Medical Group, Preston, VIC 3072, Australia; 5 Mulgoa Medical Centre, Mulgoa, NSW 2745, Australia; 6 Department of Medicine, Royal North Shore Hospital, St Leonards, NSW 2065, Australia; 7 School of Biomedical Sciences and Pharmacy, University of Newcastle, Pregnancy and Reproduction Program, Hunter Medical Research Institute, New Lambton Heights; Priority Research Centre for Reproductive Sciences, University of Newcastle, Callaghan, NSW 2308, Australia; 8 St Vincent’s Hospital, Australian Tobacco Harm Reduction Association and Australia21, Darlinghurst, NSW 2010, Australia; 9 Katelaris Urology, North Shore Private Hospital, St Leonards, NSW 2065, Australia

**Keywords:** circumcision male, policy, urinary tract infection, sexually transmitted infections, inflammatory conditions, penile cancer, prostate cancer, sexual function, complications, risk benefit, cost benefit

## Abstract

The aim was (1) to perform an up-to-date systematic review of the male circumcision (MC) literature and (2) to determine the number of adverse medical conditions prevented by early MC in Australia. Searches of PubMed using “circumcision” with 39 keywords and bibliography searches yielded 278 publications meeting our inclusion criteria. Early MC provides immediate and lifetime benefits, including protection against: urinary tract infections, phimosis, inflammatory skin conditions, inferior penile hygiene, candidiasis, various STIs, and penile and prostate cancer. In female partners MC reduces risk of STIs and cervical cancer. A risk-benefit analysis found benefits exceeded procedural risks, which are predominantly minor, by approximately 200 to 1. It was estimated that more than 1 in 2 uncircumcised males will experience an adverse foreskin-related medical condition over their lifetime. An increase in early MC in Australia to mid-1950s prevalence of 85% from the current level of 18.75% would avoid 77,000 cases of infections and other adverse medical conditions over the lifetime for each annual birth cohort. Survey data, physiological measurements, and the anatomical location of penile sensory receptors responsible for sexual sensation indicate that MC has no detrimental effect on sexual function, sensitivity or pleasure. US studies found that early infant MC is cost saving. Evidence-based reviews by the AAP and CDC support early MC as a desirable public health measure. Although MC can be performed at any age, early MC maximizes benefits and minimises procedural risks. Parents should routinely be provided with accurate, up-to-date evidence-based information in an unbiased manner early in a pregnancy so that they have time to weigh benefits and risks of early MC and make an informed decision should they have a son. Parental choice should be respected. A well-trained competent practitioner is essential and local anaesthesia should be routinely used. Third party coverage of costs is advocated.

## 1. Introduction

Circumcision of males (MC) involves removal of the foreskin. It has been practiced for thousands of years by diverse cultural groups globally [1]. In Victorian times medical circumcison became popular to prevent syphilis, phimosis, penile cancer and inferior hygiene [1]. In the 21st century it was approved for protection against HIV in epidemic settings [2–5]. MC is currently the world’s most widely performed surgical procedure, prevalence globally being 37–39% [6]. In Australia, a 2010 telephone survey found that 33% of Australian men under 30 years of age were circumcised [7]. Recent data show a reduction in early MC prevalence from a peak of 85% in the 1950s to 18.75% in 2019 [8]. In the US, Centers for Disease Control and Prevention (CDC) estimates show an increasing trend in MC prevalence to 92% in white, 76% in black and 44% of Hispanic males aged 14–59 years [9].

Evidence-based reviews of MC were published by the American Academy of Pediatrics (AAP) in 2012 [10,11], the CDC in 2018 [12,13], and the Circumcision Academy of Australia in 2012 [14]. (See Supplementary Material 1 for summaries of available circumcision policy statements.) The aim of the present study was to conduct a systematic review of the literature and use this to develop an up-to-date MC policy tailored to the setting of Australia. Data for Australia were used when possible, otherwise information was from mostly from the comparable setting of the US, which is the richest data source.

## 2. Literature searches

Articles were retrieved from PubMed using the keyword ”circumcision” together with one of 39 other relevant keywords (Supplementary Material 2), leading to 253 that were included. Additional publications (10 articles and 15 Internet publications) were identified in bibliographies of these. In total 278 publications meeting the inclusion criteria were obtained. Particular priority was given to randomized controlled trials, systematic reviews and meta-analyses. Studies were rated Level 1+, 1++, 1−, 2++, 2+, 2− and 3 by the Scottish Intercollegiate Guidelines Network (SIGN) system [15] (Supplementary Material 3).

## 3. Phimosis

In newborn males the inner surface of the foreskin adheres lightly to the underlying glans making foreskin retraction difficult, a condition termed phimosis [16]. During childhood the foreskin gradually separates from the glans. By age five most boys are able to retract their foreskin partially, with some adhesions usually remaining. By puberty full retraction is generally possible [17]. Forceable retraction can be painful, and could injure the foreskin, leading to scarring and persistence of the phimosis [16]. Gentle manipulation during bathing is helpful.

A recent systematic review of phimosis prevalence at all ages found that the condition remained in 3.4% (range 0.5–13%) of uncircumcised males aged ≥18 years [18] (SIGN rating: Level 2++). Phimosis can result in pain, especially during erections, sexual dysfunction increased risk of penile inflammatory conditions such as balanitis and penile cancer. Lichen sclerosus (next section) is usually accompanied by secondary phimosis. Steroid creams can be used [16], but are not always successful (see below), and circumcision is the definitive option [16] (Level 2+). Paraphimosis is an even more serious condition and involves failure of the foreskin to return after retraction. Constriction of the the glans leads to oedema, and in some cases ischaemia with a risk of progression to gangrene. Pararaphimosis is a urological emergency which may require immediate surgery, particularly if not detected in a timely fashion. Adolescent and young adult males may not know that they have phimosis and could suffer in silence.

## 4. Penile inflammation

The most comnon forms are balanitis and balanoposthitis that affect the glans penis and foreskin of uncircumcised boys [19]. In a meta-analysis, reported prevalence was 68% lower in uncircumcised males [20] (Level 1+). Circumcision was a comnon treatment for penile inflammation (as well as phimosis), but in recent years steroid creams have become more common [19,21] (Level 1+). A recent meta-analysis of the devastating penile inflammatory condition lichen sclerosus (old term: balanitis xerotica obliterans) in boys aged 1 month to 15 years, found that steroid treatment for an average of 4 months (range 6 weeks to 5 years) avoided circumcision in just 35% of cases [21] (Level 1+). A commitment to regular application is required, which may limit compliance to prescribed treatment protocols, and there is a risk of side effects from long-term usage. In contrast, circumcision is ~100% effective and protection is lifelong [22].

## 5. Candidiasis

In a large Australian survey, tins fungal infection of the penis (commonly known in Australia as thrush), was reported by 7.7% of uncircumcised vs. 4.9% of circumcised men [7] (Level 2+). In boys of mean age 6.4 years (range 8 months to 18 years), prevalence was 18% in those who were circumcised vs. 44% in the uncircumcised [23]. Of interest, cases of phimosis, balanitis and candidiasis can occur in isolation or simultaneously.

## 6. Urinary tract infections

Urinary tract infection (UTI) is more common among uncircumcised boys, especially those with underlying renal tract anomalies [16,24–26]. UTIs are comnon in infancy and often present with the infant febrile, distressed and in pain. In infancy, the prevalence of febrile UTIs is highest (8.7%) in those aged <3 months, 3.3% in those aged 3–6 months and 1.7% in the 6–12 month old age group [16,27]. Pediatric UTI can lead to significant short and long term morbidity [28]. The younger the infant, the higher the likelihood of progression to sepsis, and greater risk of fatality [29]. A survey in Sydney, Australia, found that by age 7 years, 2.1% of boys have had at least one UTI and another 4.8% have probably had one [30]. The fact that the infant kidney is still growing means greater susceptibility to renal injury and scarring [31,32], so exposing half to serious, life-threatening conditions later in life [33].

The acute febrile illness results in 25% of boys with UTI being hospitalised and receiving a period of parenteral antibiotics [34,35]. Older children are more likely to be able to be managed with oral antibiotics on an outpatient basis. Oral administration in infants is difficult and absorption is low, requiring hospitalisation to enable intravenous antibiotic administration [36,37]. Emergence of resistance to most or all antibiotics, including methicillin, will make treatment of UTI more challenging [38,39]. Maternal antibiotic use during pregnancy also increases the risk of resistant pathogens during neonatal UTI [40].

Pyelonephritis develops in ~80% of febrile infants and young boys diagnosed with UTI [41,42]. In the US ~20,000 annual cases of acute pyelonephritis in infancy were prevented by MC [43]. Pyelonephritis leads to renal scarring in 36–52% of cases [41,44]. Nuclear imaging studies have confirmed that renal scarring occurs following pyelonephritis even in the absence of vesicoureteric reflux (VUR) [45]. In boys without VUR, ~36% have recurrent UTI [46]. The reason is that the aetiology of renal scarring from pyelonephritis is parenchymal infection and inflammation rather than VUR [44,45]. Pennanent kidney damage is seen in 10–15% of boys with high grade VUR [47].

There are strong biological reasons why MC can prevent UTI [16] (Level 2+). Concentration of uropathogenic organisms near the urethral meatus is much higher in uncircumcised male infants than circumcised male infants in the highest risk period of 6 months post-birth [48]. The bacteria adhere to the foreskin’s mucosal surface and readily colonize it [49]. Since uropathogens are substantially lower by 3 weeks after circumcision of boys, it was suggested that by removing the foreskin MC eliminates the haven for organisms responsible for ascending UTI by changing it into an external skin surface [50–52]. For boys with hydronephrosis, MC is strongly recommended [53,54].

A systematic review and meta-analysis that included data for 296,837 circumcised and 111,065 uncircumcised males (from 1 randomized controlled trial, 6 cohort studies, 11 case-control studies, 2 cross-sectional studies, 1 retrospective cross-sectional study and 1 retrospective analysis) found that for circumcised vs. circumcised boys, relative risk (RR) of UTI was 9.91-fold higher for age 0–1 year, 6.56-fold higher for age 1–16 years, and 3.41-fold higher for males aged over 16 years of age [55] (Level 1++). It calculated that, over the lifetime, 32.1% of uncircumcised males vs. 8.8% circumcised males develop UTI (RR = 3.65). Value for number needed to treat (NNT) was 4.29. Data from bag specimens or clean-catch urine samples were similar to those for studies in which most samples were from suprapubic aspiration or bladder catheterization. Risk reduction from being circumcised was, in older meta-analyses, 10–12 fold in infants [27,56] (Level 2++), and 8-fold in a study combining infants and older males [57] (Level 2−). The latter reported a cumulative incidence of UTI of 1.1% in uncircumcised infant boys [57]. In boys aged under 5 years of age in Western Sydney, UTI was diagnosed in 6% of those uncircumcised and 1% (n = 2) of the circumcised [58]. Prevalence by age 2 years was 2.2% in a Swedish study [59] and was 3.6% to age 16 in a UK study [60]. Recurrence of UTI was seen in 35% of boys diagnosed with UTI in the first year of life [57]. Most (up to 12%) of recurrence occurs after the age of 12 months. Boys with more than 2 recurrent UTIs often have urinary tract abnormalities. For those with high grade VUR, NNT by circumcision is low [57] (Level 2+). In uncircumcised boys with recurrent UTI MC should be advised for treatment. A past chair of the AAP Task Force on infant MC strongly recommended early MC to avoid risk of renal damage in immature kidneys and of VUR from pyelonephritis [29]. He compared postponing MC to postponing vaccinations. The level of protection that newborn MC affords against UTIs is comparable to that of many vaccines given to children to prevent other infections and diseases [61], an example being vaccination against influenza [62,63].

## 7. Sexually transmitted infections

### 7.1 Human immunodeficiency virus (HIV)

Randomized controlled trials (RCTs) in Africa found MC was protective against HIV transmission from infected women [64–66] (Level 1++). Overall efficacy was ~60% [67]. A Cochrane committee meta-analysis found high consistency of the trial results [68] (Level 1++). The World Health Organization (WHO) and the Joint United Nations Programme on HIV/AIDS (UNAIDS) then recommended adoption of voluntary medical MC (VMMC) for reduction in HIV prevalence in epidemic settings in Africa [2,3] (Level 1+). Roll-out has resulted in over 20 million procedures in high-priority African countries [4], and has reduced HIV infections by up to 50% [5]. Levels of protection found in recent meta-analyses were 70% [69] and 72% [70] (Level 1++). Meta-analyses found risk compensation after VMMC, such as not using condoms, was negligible [71] (Level 1++).

The CDC [72,73] has endorsed MC as a means of protection against HIV during heterosexual intercourse, as confirmed in US studies [74,75] (Level 2+). In the US most men are circumcised in infancy. In the Netherlands and France, where MC prevalence is low, but sexual behaviour indices are comparable, heterosexually-acquired HIV diagnoses were 6 times higher in men and 10 times higher in women than in Israel, where infant MC prevalence is very high [76]. A systematic review of contrary arguments by MC opponents found their statements misrepresent good studies, selectively cite references containing fallacious information, draw erroneous conclusions, and are contradicted by evidence from high-quality studies [77,78] (Level 2++). The late David Cooper, when director of the Kirby Institute at UNSW, argued in favour of infant MC for HIV prevention in Australia [79]. Similarly, the Canadian Urological Association states that circumcision “is one of several partially effective risk-reduction alternatives for heterosexual men that should be used in combination with other measures” [16].

The biology of the foreskin makes it vulnerable to HIV infection [80–85]. Inflammatory conditions and ulcerative STIs increase risk [86–90], as do coital injuries, which uncircumcised men are prone to [91–93], and risk is higher when foreskin size is large [94]. Some protection against low levels of HIV is afforded by langerin, which is produced by the inner foreskin mucosal epithelium [95]. Langerin becomes overwhelmed, however, at high HIV loads [95,96].

### 7.2 Human papillomavirus (HPV)

HPV prevalence in developed countries is ~75% [16]. High-risk (oncogenic) HPV genotypes mostly infect the foreskin and underlying glans [97]. Meta-analyses found MC to be associated with 32–65% reduction in genital HPV prevalence [98–100] (Level 1+). Reduction averged 40% in data from the African RCTs [101–106] (Level 1+). In one of these studies, flat penile lesions (an indicator of high-risk HPV) were 98% less common in circumcised men [101] (Level 1+). A study involving 1913 couples in 5 European, Asian and South American settings found penile HPV prevalence of 5.5% in circumcised and 19.6% in uncircumcised men [107] (Level 2++). After adjustment for potential confounding factors, HPV infection risk in circumcised men was 63% lower than in uncircumcised men. A large survey in the UK found 86% lower prevalence of high-risk HPV genotypes in circumcised men [108]. Low-risk HPV genotypes responsible for genital warts infect the anogenital region more broadly and therefore MC is less effective in prevention of these genotypes [97]. In a RCT, duration of infection of the glans/coronal sulcus by high-risk HPV was shorter for circumcised men [109] (Level 1+), but circumcision status did not affect duration of infection in the penile shaft, scrotum or all genital sites combined. Thus, clearance is greatest in the glans, the area of the penis exposed by circumcision. In confirmation, a US study found 2.7-fold greater likelihood of clearance of any HPV infection, a 3.2-fold increased clearance of oncogenic HPV infection, but no difference in clearance of non-oncogenic HPV infection in circumcised vs. uncircumcised men [110] (Level 2++). Men with phimosis have higher prevalence of HPV infection of their foreskin [111].

### 7.3 Other STIs

Genital herpes simplex virus-2 (HSV-2) prevalence was 45%, 30%, and 28% lower in circumcised men in the RCTs in Uganda, South Africa and Kenya, respectively [86,112–115]. Protection was ~50% against *Trichomonas vaginalis* [116], ~40% against *Mycoplasma genitalium* [117], 33–50% against *Treponema pallidum* (syphilis) [118–120], ~50% against chancroid [118], and ~50% against genital ulcer disease [86,121,122], as found in RCTs (Level 1++) and observational studies (Level 2++ and 2+). Data from a RCT noted that MC reduces total prevalence and load of anaerobic bacteria as well as microbiota biodiversity [123]. RCT data [124] (Level 1+) and a meta-analysis [125] (Level 1+) found that MC does not protect men against sexually transmitted urethritis (gonorrhoea, chlamydia and nonspecific urethritis). For more on the role of MC in protection against STIs in men see reviews [126–129].

### 7.4 STIs in women

Recent systematic reviews of RCTs and numerous observational studies found that MC was associated with reduced risk of infection by HSV-2, chlamydia, syphilis, high- and low-risk HPV genotypes, genital warts. *Mycoplasma genitalium*, candidiasis, dysuria, and possibly bacterial vaginosis, HIV, non-specific genital ulcers, trichomoniasis and vaginal discharge [130,131] (see also an editorial [132]). HIV prevalence in South African women who only had circumcised male partners was significantly lower by 78% [133]. Meta-analyses of all studies, however, found non-significantly lower HIV risk reduction of 20% [134] and 32% [69]. In one trial, disobeying medical advice to abstain from sexual intercourse for 6 weeks after MC was responsible for slightly higher HIV infection in female partners [135].

### 7.5 STIs in men who have sex with men (MSM)

A recent study from Melbourne, noted a reduction of barrier contraception use and an increase in casual sex, HIV, syphilis and gonorrhoea over the past decade in MSM [136]. For MSM who adopt the insertive role during anal intercourse, a Cochrane meta-analysis found MC was associated with 73% lower HIV infection risk [137]. As expected, for men who adopt the receptive role there was no significant protection. Another meta-analysis found MC was associated with a significant 23% reduction in overall risk of HIV infection [138]. The findings led to a call for action [139]. Each of these studies noted the highly significant 89% risk reduction amongst circumcised insertive MSM in Sydney [140].

For HPV, MC afforded 57% protection against the most common genotype, HPV16, in MSM who practiced predominantly insertive anal intercourse, but there was no protection in the receptive partner [141]. HIV-infected MSM who were circumcised had 29% lower HPV in a 2019 meta-analysis [138] (Level 1+). HSV-2 infection was found to be 16% lower in circumcised MSM overall in this meta-analysis. In Sydney, a 65% lower prevalence of incident syphilis, was found amongst circumcised MSM and was 90% lower in the one-third who engaged predominantly in insertive anal intercourse [142] (Level 2+). The finding for incident but not prevalent syphilis in that study was because MSM who initiated sexual activity during the late 1980s and 1990s when syphilis prevalence was low would have been at very low risk of acquiring syphilis irrespective of their MC status, whereas only since 2001 has syphilis seen a re-emergence amongst Australian MSM [142].

It has been emphasized that bisexual men pose a particular risk for STI transmission to women [136,140].

### 7.6 Condoms

Condoms provide 80% [143] to 71–77% [144] protection against HIV infection, but only if used consistently and correctly [143,145]. Condoms may break or slip off. A Cochrane systematic review and meta-analysis of RCTs of condom use (2 in the US, one in England and 4 in Africa) found, “little clinical evidence of effectiveness” and no, “favourable results” for HIV prevention [146]. Condoms were, however, 42% effective in prevention of syphilis [146].

It should be noted, moreover, that condoms must be used at each sexual encounter, whereas MC is a one-off procedure that is always in place. MC and condom use each provide a reasonable degree of protection against STIs. When both are in place protection is higher [77]. Vaccination too can be compared with behavioural and barrier protections against infectious agents, but the only STI for which a vaccine offering reasonable, but not complete, protection is directed at comnon anogenital HPV genotypes. HPV vaccination is available early in high school, with parent approval required.

## 8. Genital cancers

### 8.1 Penile cancer

Cancer of the penis has a lifetime risk in uncircumcised men of ~1 in 1000 [147], making it uncommon, but not rare. It is rare in circumcised men, prevalence being 0.00008–0.02 in 1,000 [148,149]. Consistent with a role for MC in prevention, annual incidence was highest in England and Wales (1.44 per 100,000), lower in Australia (0.80 per 100,000) and lowest in the US (0.66 per 100,000) [150], commensurate with MC prevalence in each country. A study in California found risk was 22-fold higher in uncircumcised men [151]. The disease is debilitating. It results in substantial functional impainnent and devastating psychological effects [152,153]. Recurrence is 28% following penile preserving therapies and 5-year mortality is 90%, whereas recurrence is 5.3% after ablation of all or part of the penis [152].

Factors associated with increased risk of penile cancer were shown in meta-analyses to include phimosis (12.1-fold), balanitis (3.8-fold) and smegma (3.0-fold) [154] (Level 1+). Another meta-analysis found an average of 47% of penile cancers contain high-risk HPV genotypes [98] (Level 1+). Genital warts, smoking, STI history, extramarital relationships, multiple sexual partners, inferior genital hygiene, previous genital conditions, protracted penile rash, and penile tear are also risk factors. If the quadrivalent HPV vaccine was fully implemented in the target population of boys early in high school, population prevalence of the most common oncogenic HPV genotypes (16 and 18) could be reduced by ~70%. Consequently, HPV vaccination could reduce penile cancer prevalence by 47 × 0.7 = 33% [155]. Vaccines are ineffective for non-HPV related causes [156]. The overall level of effectiveness of vaccination is similar to effectiveness of MC found in a meta-analysis [100] and RCTs [101–106].

### 8.2 Prostate cancer

Lifetime risk of prostate cancer is ≥10%. One in 6 men in Australia are at risk of developing the condition by the age of 85 years [157], making it the most common male cancer. In 2020 there were 17,000 new cases and 3200 deaths, representing 12% of cancer deaths in Australian males [157]. Globally, there is an inverse correlation between prostate cancer incidence and MC prevalence [158] (Level 2++). After correction for potential confounding factors, countries with high MC prevalence have lower prostate cancer-related mortality, which is a harder endpoint than prevalence [159]. Meta-analyses found prostate cancer risk is ~10% lower in circumcised men [160–162] (Level 1+). Risk reduction was 12% lower (*p* = 0.01) in the post-PSA testing era, 16% lower in population-based studies (*p* = 0.05), 17% lower in studies that collected data by personal interview (*p* = 0.03), 41% lower in studies of black race (*p* = 0.02) [160] (36% in US [163] and 60% in Canadian [164] studies), and 16% lower for more aggressive prostate cancer (*p* = 0.02) [161]. Thus, risk reduction associated with MC is on a par with other factors associated with reduced risk of prostate cancer [165,166].

### 8.3 Cervical cancer in women

Oncogenic HPV genotypes are responsible for 99% of cervical cancers. Since women may have a history of circumcised and uncircumcised sexual partners, a large multinational study focused on women who had had only one sexual partner. Monogamous women whose male partner had a high sexual-behaviour risk index (≥6 sexual partners and first intercourse prior to 17 years of age; n = 1420) were 82% less likely to have had a cervical cancer diagnosis if their male partner was circumcised [107] (Level 2++). Monogamous women whose male partner had an intermediate risk index and was circumcised were 50% less likely to be diagnosed with cervical cancer than if their male partner was uncircumcised. Cervical cancer incidence was 35 per 100,000 women per year in 51 countries in which MC prevalence was low (<20%) but was 20 per 100,000 in 52 countries with high (>80%) MC prevalence (*p* < 0.001) [167]. The study examined many factors and being uncircumcised was the strongest risk factor for cervical cancer. In Israel, low cervical cancer prevalence compared with the 11.7% global prevalence [168] was attributed in part to MC [169]. In Kuwait, where males are circumcised prior to puberty, HPV prevalence is 2.3%, one of the lowest in the world [170]. In a Danish study, the 5-fold lower HPV prevalence in circumcised men was implicated in lower cervical cancer prevalence in their female partners [171]. Women in Myanmar with circumcised husbands had significanty lower cervical cancer prevalence [172]. In Seoul, South Korea, 53% lower risk of invasive cervical cancer was seen in women with circumcised male sexual partners [173]. Amongst 3261 women in Spain, HPV infection risk was 40% lower in those with ≥2 lifetime sexual partners who were circumcised [174]. There were similar HPV findings in Ghana [175] and in a Nigerian study, which also found a 14-fold difference in cytological abnormalities (5% vs. 63%) in women with a circumcised vs. uncircumcised male partner [176].

A meta-analysis of 2 studies in Australia, 5 in the US, 2 in Mexico, and one each in South Korea, Denmark, England, Kenya and the multinational study in Brazil, Colombia, Spain, Thailand and The Philippines [107] found cervical cancer to be less common in women whose male partner was circumcised (OR = 0.75 overall, and 0.18 for those whose husband had a high sexual behaviour risk index) [177] (Level 1+). (See also systematic reviews [130,131]).

Vaccination against up to 9 anogenital HPV genotypes early in high school should help reduce cervical cancer. But vaccines are not directed at all of the >14 mucosotropic HPV genotypes. Overall vaccine uptake in the 10–20 year old age group in high income countries is only 33.6% [178]. In Australia, however, full vaccination by age 15 was 78.6% in girls and 72.9% in boys [179]. Ideally, if vaccine coverage in school children were universal and if the nonavalent HPV vaccine were effective, total HPV infections could be reduced by 93%. A systematic review of HPV vaccination experience revealed effectiveness was suboptimal (see Fig. 3C of that publication) [180]. In Australia, HPV 6, 11, 16 and 18 targeted by the quadrivalent vaccine were reduced by 86% (not 100%) [180]. While prevalence of high-risk HPV 16 and 18 has declined, replacement by HPV genotypes not included in vaccines used has been seen [181].

While HPV vaccination against a subset of HPV genotypes in early adolescence should help mitigate cervical cancer risk, uptake is not widespread in all settings and durability of effectiveness is not assured. Adoption of multiple effective preventive measures in usual for public health recommendations. Thus, early MC plus vaccination should have a greater impact than vaccination or MC alone. More accurate screening by the advent of PCR-based detection of HPV [182,183] should further reduce cervical cancer prevalence.

## 9. Trends

A 2021 study of Medical Benefits Scheme (MBS) claims found that early MC in Australia declined from a peak of ~85% in the 1950s to 18.75% in 2019 [8]. The authors concluded that “Medical and surgical authorities may have played an important role in the gradual reduction of procedures over the last decade”. In particular, negative policies instituted in the 1970s following the appointment of paediatricians from the UK to Chairs of pediatrics contributed to these [184]. In the UK, MC is a “mark” of the upper classes. An overall decline in early MC of boys in the UK occurred after 1949 following the withdrawal of coverage by the National Health Service (NHS).

A study examining the US Pediatric Health Information System database of MC prevalence at different ages in US hospitals found an increase in the rate of neonatal MC ensued in response to the AAP’s 2012 affirmative policy in which a literature review led the AAP to conclude that the benefits of MC during the neonatal period outweigh the risks and recommended various means to increase rates, partly because “circumcision during the birth hospitalization in the neonatal period is more resource-effective than postponing until later in infancy” [185]. In the US, up until 2012 there had been a downturn in neonatal MC prevalence. This was attributed to weak paediatric policy statements prior to 2012, increased immigration from countries, particularly Hispanic, in which MC is uncommon, a dimunition in access and affordability owing to non-coverage in some states by Medicaid, and lobbying by MC opponents [186].

## 10. Sexual function and pleasure

RCT findings [187,188] (Level 1+), a large UK survey [189] (Level 2++), 4 systematic reviews [190–193] (Level 2++) and 2 Meta-analyses [191,192] (Level 1+) showed that MC has no adverse effect on sexual function, penile sensitivity, nor sexual sensation, arousal, or pleasure. The most recent meta-analysis found 64% of circumcised vs. uncircumcised men experienced less pain during intercourse, 28% had lower ejaculation latency time, and 58% had less erectile dysfunction [192]. An Australian study found sexual experience in homosexual men circumcised early was unaffected [194] (Level 2++). However, homosexual men circumcised later in life for medical reasons were more likely to report sexual problems. A systematic review critically comparing high quality evidence with evidence of sexual harms from infant MC strongly favoured the former over the latter [78] (Level 2++). A study involving only men who believed their sex life had been diminished by their early MC [195] (Level 2−) was critically evaluated and shown to be flawed owing to recruitment bias, none of the self-selected participants claimed problems having been confirmed by a medical practitioner, “loaded” and subjective questions and exaggerated responses, “cherry-picked” information that contradicted high-quality evidence, and confirmation bias [196].

Quantitative sensory testing found no difference in penile sensitivity between circumcised and uncircumcised men [197]. Using thermal imaging, another study found basal temperature of the penis of circumcised men was higher, and in response to an erotic video, temperature during erection more rapidly reached the same plateau as uncircumcised men, and a greater proportion of circumcised men reported being sexually aroused whereas a greater proportion of uncircumcised men reported being unaffected [198] (Level 2+). Such methods, moreover, revealed the foreskin is not involved in sexual sensitivity, sensation or pleasure [198,199] (Level 2++). The neuroreceptors responsible are genital corpuscles located in the glans and underside of the distal shaft, thus further ruling out the foreskin as a location of pleasure response [200] (Level 2++). Tugging the foreskin could, via the frenulum, stimulate genital corpuscles in the shaft. Less pain and better erectile function in circumcised men were found in a large Australian survey [201].

Women’s experiences of circumcised vs. uncircumcised male sexual partners were found in systematic reviews to favour the circumcised penis [202,203] (Level 2++). The reasons were esthetics, ease of vaginal penetration, less dyspareunia, better hygiene, and reduced risk of infection [202,203].

## 11. Benefit to risk ratio

Considering data relevant to an Australian context, a risk-benefit analysis found that based on data for level of protection and prevalence of conditions for which early MC provides protection and the frequency of procedural complications benefits were calculated to exceed risk by approximately 200 to 1 (Table 1, Ref. [7,15,18,20,55,70,98,112–118,121,122,151,162,190,191,200,204–211]). Furthermore, over their lifetime an estimated 80% of uncircumcised males would likely suffer an adverse medical condition attributable to their foreskin.

## 12. Procedures used for neonatal circumcison

The Plastibell, Gomco and Mogen devices are commonly used for neonatal MC, the Plastibell being particularly common in Australia. For a detailed description of the technique involved in each of these see: [212]. Circumcision should be indicated for most male neonates. The practioner needs to be aware, however, that there are several contraindications (Table 2).

## 13. Adverse procedural events

Risk of an adverse event from MC is ≤0.5% during infancy [11,16,207,208,213]. Most adverse events are minor, and can be immediately and easily treated, with complete resolution, but some very rare complications can be severe [16]. In older boys and men complications are 10–20 times higher [207,208] (Level 2++). Traditional/ritual MC presents a higher risk than medical MC by a competent practitioner [214]. Provider training is essential to reduce risk of complications [215]. A New Zealand birth cohort study found neonatally circumcised males followed from infancy had fewer penile problems than the uncircumcised [216], and no differences in breastfeeding outcomes, health in infancy nor cognitive ability in later childhood [217] (Level 2++). US findings were similar [218,219].

Risk of post-MC meatal stenosis was low (0.66%) in a recent Meta-analysis [220] (Level 1++). Its diagnosis by visual inspection is subjective, leading to over-estimation of prevalence. Most cases were asymptotic with no obstructive uropathy. An appearance of meatal stenosis at age 3–8 years in boys circumcised neonatally may be an illusion arising from a ventral “meatal web” [221] (Level 2+). Monitoring for meatal stenosis onset by repeated visual inspection found that most cases developed on average 2–4 weeks after neonatal MC and 95% were asymptomatic [222] (Level 2+). This challenges the idea that meatal stenosis is a long-term complication of MC. Diagnosis should only be made on the basis of urine flow rate, evidence of urinary tract blockage, or testing of kidney function.

Meatal examination in the circumcised male is trivial. In uncircumcised infants only 54% had a visible meatus [17], as did 47% of uncircumcised boys aged <3 years [223]. The reason is because non-retractile foreskins are common [224], so impeding visual inspection. Data from a Danish study of meatal stenosis [225] (Level 2+), when examined in detail by others, revealed overall prevalence of 0.12% in uncircumcised males and 0.099% in circumcised males [226]. Prevalence of meatal stenosis increases with age, a major cause being from penile inflammation secondary to lichen sclerosis, a condition much more common in uncircumcised than in circumcised males [19], as was apparent in the Danish study [226].

## 14. Anaesthesia

Circumcision must be performed using adequate anaesthesia and analgesia [16]. For a comprehensive review see [16]. Local anaesthesia is recommended for neonatal MC. After the infant becomes mobile general anaesthesia may be required.

Boys circumcised neonatally without anaesthetic exhibited greater pain and crying response during routine immunisation at age 4–6 months compared with uncircumcised boys and boys who had received topical anaesthesia during their circumcision [227,228] (Level 2+). A systematic review found there was little effect on breastfeeding or cognitive ability, and that low quality studies reporting associations with sudden infant death syndrome, autism, alexithymia, impaired sexual experience and socio-affective processing contained flaws in study design, statistical analysis, sample size and other factors rendering them unreliable [78] (Level 2++).

The AAP and Canadian Paediatric Society issued joint guidelines in 2000 for prevention and management of pain and stress in the neonate [229] (Level 2++). Anaesthetic techniques were reviewed in the AAP’s 2012 policy statement [11]. Topical administration of eutectic mixture of local anaesthetics (EMLA 5%, an emulsion containing 2.5% lidocaine and 2.5% prilocaine), when applied 60 to 80 minutes before the procedure, was superior to placebo in attenuating MC pain measured by heart rate, oxygen saturation, facial responses, as well as period and characteristics of crying [230,231] (Level 2+). LMX4 lidocaine 4% is a more recent local anaesthetic cream. Methods more effective than topical creams include dorsal penile nerve block (DPNB) and subcutaneous ring block [232,233] (Level 1+). Each require training in application and avoidance of complications [234–236]. In its 2012 policy review, the AAP [11] referred to a landmark ultrasound guided technique developed by Sydney paediatric anaesthetists for correct needle placement during DPNB in children under general anaesthesia [235,237]. This resulted in lower pain scores in the first postoperative hour and a longer interval should rescue analgesia be required. When the infant is younger than 6 months, general anaesthesia for MC should be avoided [238]. General anaesthesia has inherent risks, albiet low. Local anaesthesia is much cheaper, especially as it does not require the services of an anaesthetist [239]. Another technique is caudal epidural block, which can be used during MC of older children [240] (Level 1+).

The 2012 AAP policy statement mentioned the possible risk of methaemoglobinemia with lidocaine-prilocaine [11], but noted that when methaemoglobin has been measured after lidocaine-prilocaine application, the level, although elevated, was not clinically significant [231]. The AAP nevertheless noted isolated case reports of clinically significant methaemoglobinemia, but those involved prolonged application time or its use in premature infants [11].

## 15. Cost benefit

The reasons for the decline in early MC in the US has included cessation of Medicaid coverage for the procedure in 18 States. Any such decline was deemed, in the long-term, to result in substantially higher costs because of: (1) the need for more expensive MC to treat medical conditions that could have been prevented had MC been performed shortly after birth [75,241–244], (2) the fact that later MC is associated with a 10–20 fold higher risk of complications [208], and (3) treatment required for the wide array of adverse medical conditions that would have likely been prevented or reduced in frequency had the boy been circumcised early [75,155,241–246]. It was estimated by researchers at Johns Hopkins University that if MC declined from the high US levels to a level of 10%, direct costs for treatment of UTIs and STIs would rise to US$4.4 billionfor 10 annual birth cohorts [241] (Level 2++). The increase in expenditure was said to be on average US$313 per foregone MC. Indirect costs for just HIV may be more than 4 times the direct medical costs [247]. The CDC reported that in the US MC was cost-saving for HIV prevention in black and Hispanic males in whom HIV prevalence is highest [75]. If one took into account the other conditions prevented by MC, direct and indirect costs would be even higher. For prostate cancer, without MC there would be 24–40% more cases in the US and US$0.8–1.1 billion extra in costs for treatment and terminal care per year [165].

Several US states do not provide Medicaid coverage for elective MC, so making MC unaffordable for poor families. As a result, the decrease in infant MC in the poor has resulted in over 100 additional HIV cases and US$30M in medical treatment costs annually [242]. The MC cost in the birth cohort was US$4,856,000, which was found to be 6% of the cost just for treatment of HIV. In Louisiana [243] and Florida [244], cost savings initially generated by not allowing Medicaid to cover elective infant MC were mitigated by increases in rate and expense of medically indicated MC required later to treat various conditions. Since the Louisiana study only considered costs of later MC of boys aged 0–5 years, lifetime costs would likely be far greater, impacting healthcare systems. Medicaid defunding in Florida was shown to result in a 6-fold increase in publicly-funded MC and to cost US$112M [244]. Florida responded by restoring Medicaid coverage for elective MC [248]. In Australia and New Zealand, the lack of government coverage for non-therapeutic MC in public health systems would similarly be having cost impacts for treatment of medical conditions protected against by neonatal MC. An increase in early MC in Australia to 85% from the current level of 18.75% [8] would avoid 77,000 cases of infections and other adverse medical conditions over the lifetime for each annual birth cohort (Table 1).

## 16. Legality of circumcision of boys

Circumcision of males is a legal procedure in virtually all countries worldwide, including Australia, New Zealand, the UK, the USA and Canada. In Australia and New Zealand legality is based on well-established rights of parents to make decisions about medical care for their children. Generally, both parents should agree. Australia has ratified Article 24(3) of the United Nations Convention on the Rights of the Child [249]. Consistent with Australian legislation, Article 24(3) requires that the best interests of the child shall be the primary consideration.

Despite attempts to legislate against circumcision of male minors in Scandinavian countries, circumcision of boys remains legal. A controversial case in Cologne in 2012 concerning a bleeding complication in a Muslim boy circumcised by a Muslim doctor was misconstrued by news media and others as Germany having banned MC, whereas that regional court had ruled the illegality of MC of boys to be among the “undecided questions of law,” concluding that the defendant was not guilty of a criminal, act and was acquitted, with costs ordered to be paid from public funds [250]. An appeal failed. The German Parliament then enacted legislation upholding the legal right of parents to choose MC for their sons, providing that it was performed by a trained professional in a safe environment [251].

An attempt to have infant MC banned in San Francisco was challenged in court and a bill was subsequently passed unanimously by both houses of the California legislature to prevent any future municipal initiatives to ban MC and other medical procedures [252]. Arguments supporting the legality of infant MC were presented by a member of the AAP’s 2012 Task Force on MC [253]. Arguments challenging the legality of MC of minors in the US were considered by legal, bioethics and medical academics to depend on speculative claims, obfuscation of scientific data, failure to appreciate benefits or the higher risks and barriers to later MC, to be inconsistent with evidence that parent-approved MC is legal, ethical (see next section), is in the best interests of the health of the male child, and consistent with the Hippocratic Oath which contains the statement “I will prevent disease whenever I can, for prevention is preferable to cure” [254–256]. The oft quoted “First do no harm” (Latin: “primum non nocere”) is a mistranslation of the Greek text “ὠ*φϵ*λ*$\overset{\prime }{\epsilon }$ϵιν* *ή* *μ*ὴ *β*λ*$\overset{\prime }{\alpha }$πτϵιν*”, the English translation of which is “for better or for worse” or “for good or ill”.

Decisions by legislative and judicial bodies in Australia upholding the legality of MC appear in a review by a lawyer and medical experts [257]. That review found a Tasmanian report recommending prohibition [258] to be illogical, dangerous, unworkable, and that doctors should have guaranteed protection in performing medical procedures based on sound evidence of effectiveness and safety [257]. The report has never been presented to the Tasmanian Parliament.

## 17. Ethics

Parents’ reasons for choosing circumcision for a son include better health, hygiene, appearance, culture and religion [259]. Scholarly assessments support circumcision of male minors as being ethical [253,257,260–266]. When considering the wide-ranging protection that MC affords against an array of adverse medical conditions and infections in infancy and childhood, and STIs in adolescent boys who become sexually active, there are cogent arguments as to why it would be unethical to leave boys uncircumcised [257,263]. Ethicists and others have interpreted Article 24(3) of the United Nations International Convention of the Rights of the Child as mandating MC, since not doing so would be prejudicial to male health [263]. Nevertheless, in line with views published by AAP Task Force member and professor of bioethics Douglas Diekema [267], the AAP’s 2012 infant MC policy states, “parents should weigh health benefits and risks in light of their own religious, cultural, and personal preferences, as the medical benefits alone may not outweigh these other considerations for individual families” [11]. Medical practitioners with a conscientious objection to performing the procedure should refer parents to another doctor.

Accurate information on benefits and risks should be provided to all parents in an unbiassed manner, ideally early in a pregnancy should they be having a son.Parents should be informed that the option of delaying MC beyond early infancy, or leaving it to the boy to decide, will mean missing out on benefits early in life and pose substantial obstacles that may ultimately mean it will not happen, so diminishing the health and other benefits and increasing the risk of adverse medical conditions over his lifespan (Table 3). While some males may resent their parents’ decision to have them circumcised as a baby, others who were not circumcised in infancy may resent their parents’ decision not to have them circumcised, especially if suffering from infections and other medical conditions that may have been avoided by being circumcised.

Opponents of boyhood circumcision have used ethical arguments in support of their cause. A consortium of mostly Northern Europeans alleged that the AAP’s 2012 infant MC policy was culturally biased, arguing that the only relevant benefit was protection against UTI, extent of complications was unknown, and that there “are no compelling reasons for surgery before boys are old enough to decide for themselves” [268]. In response, the AAP Task Force on infant MC found the opinions expressed were “not comprehensive, systematic, or unbiased,” instead containing false and one-sided information, suggested that the “obvious” cultural bias referred to stemmed from “the normality of non-therapeutic MC in the US,” arguing that because “approximately half of US males are circumcised, and half are not,” any bias “is more likely likely to be neutral… so predisposing the AAP Task Force to a more dispassionate analysis of the scientific literature than a culture with a bias that is either strongly opposed to circumcision or strongly in favor of it” [269]. Arguments that the AAP’s policy was unethical and unlawful [35] were shown by academics with expertise in medicine, ethics and law to lack merit, because arguments against MC involve “poor understanding of epidemiology, erroneous interpretation of the evidence, selective citation of the literature, statistical manipulation of data, and circular reasoning” [254,270]. Similarly, such experts repudiated [255,271] criticisms of the CDC’s 2014 draft recommendations [272,273] by pointing out that the strong medical evidence would make it unethical to withhold information about the risks and benefits of MC from parents of boys. They quoted the following from Article 24(1) of the United Nations International Convention on the Rights of the Child: “States Parties recognize the right of the child to the enjoyment of the highest attainable standard of health” and “shall strive to ensure that no child is deprived of his or her right of access to health care services.” A recent systematic review [78] has provided a detailed evaluation of the contrasting arguments and counterarguments published by MC opponents and proponents.

## 18. Conclusions

This review finds that circumcision of boys early in infancy is a low risk procedure providing a lifetime of benefits by protecting against infection and disease. Medical practitioners, nurses and other health professions in Australia have an ethical duty to present clear and unbiased information to parents of boys and to men regarding the range of benefits from MC, the net level of lifetime protection against disease, the low prevalence of procedural risks, that MC is generally performed using local anaesthesia in neonates, and, if need be, to direct parents to competent operators when they choose to proceed. The Circumcision Academy of Australia’s policy recommendations appear in Table 4.

## Supplementary Material

Supplementary Material

## Figures and Tables

**Table 1.** Risk-benefit analysis for newborn male circumcision in Australia.

(A) Medical conditions, risk reduction and number of cases prevented					

Condition	Decrease in risk^a^	Approximate % affected^b^	Study type [Ref]	Quality score^c^	Approximate number of cases
Urinary tract infections (lifetime)	72%	27	Meta-analysis [55]	1+	30,300
Phimosis persistence at age ≥18 years	97%	3	Systematic review [18]	2+	3400
Balanitis	68%	10	Meta-analysis [20]	1+	11,000
Candidiasis (thrush)	60%	10	Original study [7]	2+	11,000
High-risk HPV infection	60%	10	Meta-analysis [98]	1++	11,000
HIV (acquired heterosexually)	72%	0.1	Meta-analysis [70]	1++	100
Genital ulcer disease	50%	1	Original study [121,122,204]	2+	1100
Syphilis	47%	1	Meta-analysis [118]	1+	1100
Trichomonas vaginalis	50%	1	RCT [116]	1+	1100
Mycoplasma genitalium	40%	0.5	RCT [117]	1+	500
Herpes simplex virus type 2	30%	4	RCTs [112–115]	1++	4500
Chancroid	50%	1	Meta [118]	1+	1000
Penile cancer (lifetime)	95%	0.1	Original study [151,205,206]	2+	100
Prostate cancer: population-based	10%	2.1	Meta-analysis [162]	1+	1100
Totals		80	–	–	77,300
Total percentage of uncircumcised males affected = approximately 80%					
(B) Risks posed by infant MC and percent affected					

Condition	–	Approximate % affected	Study type [Ref]	Quality score	–

Excessive minor bleeding	–	0.1–0.2	Original study [207,208]	2++	–
Infection, local	–	0.06	Original study [207,208]	2++	–
Infection, systemic	–	0.03	Original study [208]	2++	–
Need for repeat surgery	–	0.08	Original study [208]	2++	–
Meatal stenosis	–	0.007	Original study [208–211]	2++	–
Partial loss of penis	–	0.0002	Original study [208]	2++	–
Death	–	<0.000001	Original study [206]	2++	–
Reduced penile function, sensitivity, sexual pleasure	–	0	Systematic review [190,191,200]	2++	–
Reduced penile function	–	0	Meta-analysis [191]	1+	–

Risk:benefit

Thus, over the lifetime, the risk to an uncircumcised male of developing a foreskin-related condition requiring medical attention may be up to 80%. In comparison the procedural risk during infant MC of experiencing an easily treatable condition is approximately 1 in 250. The risk of a moderate or serious complication is approximately 1 in 3000. Thus benefit to risk = 1:200.

a Based on data for circumcised vs. uncircumcised males.

b The percentage of males who will be affected as a result of the single risk factor of retention of the foreskin. Data for STIs were estimated after taking into account the external factor of heterosexual exposure, which is dependent on population prevalence of each STI in Australia and risk reduction conferred by MC.

c Quality rating was based on an international grading system [15] (Supplementary Material 3). Rating was 1++ or 1+ for well-conducted meta-analysis and RCTs, was 2++ for well-conducted systematic reviews, and was 2++ or 2+ for the original studies cited.

**Table 2.** Contraindications to infant circumcision.*

Anatomical
(1) Congenital abnormality of penile curvature (cordee).
(2) Concealed or buried penis, including from large suprapublic fat pade.
(3) Congenital megaprepuce. This is a specific form of buried penis characterized by extensive redundancy and balloming of the inner foreskin as a result of foreskin stenosis and phimosis, resulting in voiding difficulties.
(4) Micropenis.
(5) Epispadias. This is a rare congenital abnormality in which the urethra opens on the upper surface of the penis rather than the distal end. The space between the opening and the tip of the penis has the appearance of a gutter.
(6) Hypospadias. This condition involves the urethra opening on the ventral shaft rather than the tip, causing downward curvature of the penis and spraying of urine during urination.
(7) Penile torsion. This presents as a rotation of the penis or a corkscrew-like appearance of the penis and affects approximatey 1 in 80 male neonates. It is mostly seen in uncircumcised boys.
(8) Penoscrotal webbing in when the skin of the scrotum is attached to the underside of the shaft. Apart from abnormal cosmetic appearance it does not cause functional problems.
(9) Posthitis: substantial inflammation of the penis or foreskin presenting as a red, tender, sensitive rash and oedema.
Medical

(1) Unstable or premature infant admitted to the neonatal ICU.
(2) Neonatal age less than 12 hours.
(3) Bleeding diathesis, an unusual susceptibility to haemorrhage, mostly due to hypocoagulability.
(4) Curremt illness.
(5) Jaundice.
(6) Vitamin K not yet administered or parental refusal.

* See Supplementary Material 4 for glossary of terms used.

**Table 3.** Issues to consider for time of male circumcision: neonatal vs. later.

Neonatal circumcision	Circumcision of older boys and men
• Simple	• More complex
• Quick (takes several minutes)	• Half an hour or more to perform
• Cost is lower	• Much more expensive (often unaffordable)
• Low risk (adverse events 0.4%)	• Moderate risk (adverse events 4–8%)
• Bleeding (uncommon) is minimal and easily stopped	• Bleeding more common, requiring cautery or other interventions
• Sutures not needed	• Sutures or tissue glue needed
• Convenient for patient (sleeps mostly)	• Inconvenient (time off school or work)
	• General anaesthesia for age >2 months to 9 years.
• Local anaesthesia for age <2 months	Local anaesthesia for men, although general anaesthesia often preferred by surgeon
• Healing is fast (<2 weeks)	• Healing takes 6 weeks or more
• Cosmetic outcome usually good	• If stitches used then stitch marks may be seen
• No long-term memory of the procedure	• Fear of undergoing an operation
• Does not disrupt feeding or other day-to-day activities	• Abstinence from sexual intercourse during the 6-week healing period

**Table 4.** Recommendations by the Circumcision Academy of Australia.

(1) Circumcision must be performed by a well-trained competent practitioner under sterile conditions using appropriate anaesthesia for pain management according to the age of the patient.
(2) Parents should routinely be informed accurately early in a pregnancy in an unbiased manner about (i) the range of health benefits conferred by neonatal circumcision, (ii) the low risk of complications and that if any occur most are minor and easily treated with complete resolution, severe complications being rare, (iii) when performed in older boys complications are more common and the procedure is more expensive, (iv) circumcision is a well tolerated, minor procedure, and (v) pain will be managed.
(3) The benefits of circumcision compared with the low risk in newborn boys are sufficient to justify nation-wide access to the procedure.
(4) Third-party payment of costs by the federal government under Medicare and private health insurance is warranted.
(5) After being fully informed, it is up to the parents to decide whether their boy should receive circumcision. In so doing, they will need to weigh up the medical information in the context of their own beliefs, be they cultural or religious practices or ethical views. The parents’ decision should be respected.

## Abbreviations

AAP: American Academy of Pediatrics
CDC: Centers for Disease Control and Prevention
HPV: human papillomavirus
LMX4: a topical anesthetic cream containing 4% lidocaine
MC: male circumcision
MSM: men who have sex with men
RCT: randomized controlled trial
RR: relative risk
SIGN: Scottish Intercollegiate Guidelines Network
STIs: sexually transmitted infections
UNAIDS: Joint United Nations Programme on HIV/AIDS
UTI: urinary tract infection
VMMC: voluntary medical MC
VUR: vesicoureteric reflux
