# Supplementary material for: Evidence-based circumcision policy for Australia
Source: J Mens Health. Author manuscript; Available in PMC 2022 Aug 25. (PMC9409339; doi:10.31083/j.jomh1806132)
Supplement: Supplementary Material [file NIHMS1830279-supplement-Supplementary_Material.pdf]

Supplementary Material to:

Evidence-based circumcision policy for Australia

Supplementary Material 1. Summary of policy statements of other medical organizations

American Academy of Pediatrics (AAP) 2012 [2,3] (Quality level 2++)

Systematic evaluation of English-language peer-reviewed literature from 1995 through 2010 indicates that preventive health benefits of elective circumcision of male newborns outweigh the risks of the procedure.

Benefits include significant reductions in the risk of urinary tract infection in the first year of life and, subsequently, in the risk of heterosexual acquisition of HIV and the transmission of other sexually transmitted infections.

The procedure is well tolerated when performed by trained professionals under sterile conditions with appropriate pain management. Complications are infrequent; most are minor, and severe complications are rare. Male circumcision performed during the newborn period has considerably lower complication rates than when performed later in life.

Although health benefits are not great enough to recommend routine circumcision for all male newborns, the benefits of circumcision are sufficient to justify access to this procedure for families choosing it and to warrant third-party payment for circumcision of male newborns. It is important that clinicians routinely inform parents of the health benefits and risks of male newborn circumcision in an unbiased and accurate manner.

Parents ultimately should decide whether circumcision is in the best interests of their male child. They will need to weigh medical information in the context of their own religious, ethical, and cultural beliefs and practices. The medical benefits alone may not outweigh these other considerations for individual families.

Findings from the systematic evaluation are available in the accompanying technical report. The American College of Obstetricians and Gynecologists has endorsed this statement.

Centers for Disease Control and Prevention (CDC) 2018 (Quality level 2++)

Document 1:

Information for providers counseling male patients and parents regarding male circumcision and the prevention of HIV infection, STIs, and other health outcomes: Aug 22, 2018. https://stacks.cdc.gov/view/cdc/58456 [4].

(Section 4 below pertains to paediatric circumcision)

4. ***Providing information to parents of male newborns, children, or adolescents***

Health benefits and risks of elective neonatal, pediatric, or adolescent male circumcision should be considered in consultation with medical providers. Ideally, discussions about neonatal circumcision should occur prior to the birth of the child. Ultimately, whether to circumcise a male neonate or child is a decision made by parents or guardians on behalf of their newborn son or dependent child. When providing information to parents about male circumcision for an adolescent minor, the adolescent should be included in the decision-making process about undergoing elective male circumcision. When providing information to an adolescent inquiring about male circumcision, parents should be engaged in the discussion, unless the adolescent is legally emancipated. Minors may be deemed emancipated, giving them sole authority to make health care decisions on their own behalf under certain circumstances, which vary by state law; for example, if the minor 1) lives independently and is self-supporting, 2) is married, 3) is pregnant or a parent, 4) is in the military, or 5) is declared emancipated by a court as defined in the mature minor section.

4-A. ***Parents and guardians should be informed about the medical benefits and risks of neonatal, pediatric, or adolescent medically performed male circumcision (see Box 1):***

• During infancy, circumcised infants are less likely than uncircumcised infants to experience urinary tract infections (UTIs); an estimated 7% of infant males presenting with fever in outpatient clinics and emergency rooms had UTIs, including 20% of uncircumcised febrile infants and 2% of circumcised febrile infants aged younger than 3 months of age.
• An estimated 32% of uncircumcised males compared with 9% of circumcised males will experience a UTI in their lifetime, suggesting that circumcision is associated with a 23% absolute decreased lifetime risk of UTI.
• Although most UTIs are treatable, serious complications may occur when UTIs are not diagnosed, recurrent, difficult to treat, or left untreated. Such complications may include sepsis, pyelonephritis, and renal scarring and have been associated with an increased risk for long-term consequences, including hypertension, build-up of kidney waste products (uremia), and end-stage renal disease.
• An estimated 14% of uncircumcised boys compared with 6% of circumcised boys experienced balanitis, irritation, adhesions, phimosis or paraphimosis, suggesting that circumcision is associated with an 8% absolute decreased risk of these conditions.
• During adulthood, circumcised males were less likely than uncircumcised males to experience penile cancer.
• Other anticipated health benefits derive in part from future prevention of HIV and some

STIs acquired through heterosexual sex. Eight percent of annual HIV diagnoses in the United States are among persons with infection attributed to heterosexual contact. STIs are very common, with human papilloma virus (HPV) infection of the anus or genitals occurring in many sexually active persons, although HPV vaccination is highly effective against many serotypes. Current risks for either HIV or other non-HIV STIs may not remain constant in the future and the future risk for any individual neonate, child, or adolescent cannot be definitively defined at the time that a circumcision decision is made.
• Considerations for the timing of male circumcision:

– Neonatal male circumcision is safer, less expensive, and heals more rapidly than circumcision performed on older boys, adolescent males, and men.

– Most of the health benefits of male circumcision occur after sexual debut (i.e. after becoming sexually active).

– Male circumcision can also be conducted in adulthood when the individual can make the decision for himself. However, male circumcision after sexual debut could result in missed opportunities for:

HIV and STI prevention during the window period between sexual debut and circumcision

Prevention of UTIs during infancy.

• Complications of medically performed male circumcision in the United States are typically uncommon and easily managed. Severe complications are rare in all age groups and occur in 0.23% of all circumcised males overall.

–Among newborns and children aged 1–9 years, most frequently reported complications include bleeding and inflammation of the penis or incomplete wound healing or adhesions requiring corrective procedures.

Complications occur in 0.2% of infants aged ≤ 1 month,25-27 0.4% of infants aged < 1 year,24 and approximately 9% in children aged 1–9 years.

–Among persons aged 10 years and older, the most frequently reported complications include those complications reported in younger children as well as wounds of the penis.c Complications occur in approximately 5% of persons in this age group.

–There are not specific data about the frequency of complications in the adolescent age group (13–18 years).

• The American Academy of Pediatrics Taskforce on Circumcision states that the health benefits of newborn male circumcision outweigh the risks and that the benefits of newborn male circumcision justify access to this procedure for families who choose it.

4-B. Medically performed neonatal, pediatric, or adolescent male circumcision should be done by trained clinicians using appropriate (or standard) infection control, analgesia, and anesthetic practices.

Document 2:

Background, Methods, and Synthesis of Scientific Information Used to Inform "Information for Providers to

Share with Male Patients and Parents Regarding Male Circumcision and the Prevention of HIV Infection, Sexually Transmitted Infections, and other Health Outcomes" Aug 22, 2018. https://stacks.cdc.gov/view/cdc/58457 [5].

(Document 2 comprises an extensive review of the medical literature on male circumcision spanning 82 pages, with 389 references.)

Page 30, para 1, lines 6–13 states: "In a comprehensive risk-benefit analysis of infant male circumcision based on reviews of the literature and meta-analyses, it is estimated that over a lifetime, benefits exceed risks by a factor of 100:1. Based on a meta-analysis of 22 studies, most of which were conducted in the United States, it is estimated that 32.1% (95% CI = 15.6–49.8) of uncircumcised men compared with 8.8% (95% CI = 4.15–13.2) of circumcised men will experience a UTI in their lifetime, suggesting that lack of circumcision is associated with a 23.3% increased risk of UTI during a man's lifetime."

Document 3:

Peer Review Comments and CDC Responses for *Information for Providers Counseling Male Patients and Parents Regarding Male Circumcision and the Prevention of HIV infection, Sexually Transmitted Infections, and other Health Outcomes and *Background, Methods, and Synthesis of Scientific Information Used to Inform "Information for Providers to Share with Male Patients and Parents Regarding Male Circumcision and the Prevention of HIV Infection, Sexually Transmitted Infections, and other Health Outcomes"*** https://www.cdc.gov/hiv/pdf/risk/MC-HISA-Round-1-Peer-Review-Comments-and-Responses.pdf [6].

This document presents a compilation of all the comments received and the corresponding CDC responses during the first peer review comment period for a) *Information for Providers Counseling Male Patients and Parents Regarding Male Circumcision and the Prevention of HIV infection, Sexually Transmitted Infections, and other Health Outcomes* and b) *Background, Methods, and Synthesis of Scientific Information Used to Inform "Information for Providers to Share with Male Patients and Parents Regarding Male Circumcision and the Prevention of HIV Infection, Sexually Transmitted Infections, and other Health Outcomes"*

Document 4:

https://www.cdc.gov/hiv/pdf/risk/MC-HISA-Public-Comments-and-Responses.pdf

Summary of Public Comments and CDC Responses to Public Comments for *Information for Providers Counseling Male Patients and Parents Regarding Male Circumcision and the Prevention of HIV infection, Sexually Transmitted Infections, and other Health Outcomes* [7].

This document presents a compilation of all the comments received and the corresponding CDC responses during the public comment period for the document *Information for Providers Counseling Male Patients and Parents Regarding Male Circumcision and the Prevention of HIV infection, Sexually Transmitted Infections, and other Health Outcomes*

Canadian Paediatric Society (CPS) 2015 [8] (Quality level 2–)*

Abstract: The circumcision of newborn males in Canada has become a less frequent practice over the past few decades. This change has been significantly influenced by past recommendations from the Canadian Paediatric Society and the American Academy of Pediatrics, who both affirmed that the procedure was not medically indicated. Recent evidence suggesting the potential benefit of circumcision in preventing urinary tract infection and some sexually transmitted infections, including HIV, has prompted the

Canadian Paediatric Society to review the current medical literature in this regard. While there may be a benefit for some boys in high-risk populations and circumstances where the procedure could be considered for disease reduction or treatment, the Canadian Paediatric Society does not recommend the routine circumcision of every newborn male.

*See critiques [9,10] which explain why this position statement was assigned a low quality rating.

British Medical Association 2019 [11] (Quality level 2–)†

Non-therapeutic male circumcision (NTMC) of children – a practical guidance for doctors

Circumcision of male children including those who are competent, for non-therapeutic reasons, is a controversial area and a wide spectrum of views on circumcision is found within society and within the BMA's membership. For example, there are differing views over whether it is a beneficial, neutral or harmful procedure, and whether it should ever be done on a child who is not capable of deciding for himself or undertaken by non-medical practitioners.

A wide spectrum of views can also be found in men who have undergone non-therapeutic male circumcision (NTMC) themselves as children – some feel aggrieved that they were circumcised before they could decide for themselves, whereas others are pleased if, for example, they believe it is an important part of their identity and/or religion, with many going on to arrange the circumcision of their own children.

The BMA has never taken a position in the debate about the acceptability or otherwise of NTMC. Instead, as with other procedures involving children who lack the capacity to consent, we have made clear that those wishing to authorise the procedure for their children need to demonstrate that it is in the child's best interests.

Our guidance focuses on providing practical advice for doctors, including the professional standards expected of doctors performing the procedure, good practice guidelines and safeguards, and the type of factors that might be relevant in an assessment of "best interests" in this context.

The guidance is not intended to be a comprehensive detailed reflection on all the international and UK debates on the issue. As noted earlier, the guidance is primarily practical, although it also highlights, in brief, some of these debates, to illustrate the diversity of views and the context in which doctors will be making these decisions.

Our guidance does not cover circumcision carried out by non-doctors. We note that there is no requirement in law for these practitioners to have proven expertise, although there are standards that some practitioners ascribe to set by external collectives, associations and societies.

There have been rare cases in the UK where non-doctor practitioners have been imprisoned due to gross failings in the way the circumcision has been carried out, resulting in the death of, or life-changing injuries to a child. We urge parents who are considering having their child circumcised, to ensure that the practitioner who carries out the circumcision has undergone relevant training and has proven experience and competence in the practice.

†See critique published in the Royal College of Paediatrics and Child Health's journal, Paediatrics and Child Health (UK) [12].

Royal College of Surgeons of England 2000 [13] (Quality level 2–)

Statement from the British Association of Paediatric Surgeons, the Royal College of Nursing, the Royal College of Paediatrics and Child Health, The Royal College of Surgeons of England and the Royal College of Anaesthetists.

This statement refers to circumcision in male children only. Female circumcision is prohibited by law in the Prohibition of Female Circumcision Act 1995. Circumcision for religious reasons is outside the remit of this statement.

Natural history of the foreskin

- The foreskin is still in the process of developing at birth and hence is often non-retractable up to the age of three years.
- The process of separation is spontaneous and does not require manipulation.
- By three years of age, 90% of boys will have a retractable foreskin.
- In a small proportion of boys this natural process of separation continues to occur well into childhood.

Indications for circumcision

- The one absolute indication for circumcision is scarring of the opening of the foreskin making it nonretractable (pathological phimosis). This is unusual before five years of age.
- Recurrent, troublesome episodes of infection beneath the foreskin (balanoposthitis) are an occasional indication for circumcision.

• Occasionally, specialist paediatric surgeons or urologists may need to perform a circumcision for some rare conditions.

Criteria to be fulfilled in performing circumcision

• The operation should be performed by, or under the supervision of, doctors trained in children's surgery.

• The child must receive adequate pain control during and after the operation.

• The parents and, when competent, the child, must be made fully aware of the implications of this operation as it is a non-reversible procedure.

• This operation must be undertaken in an operating theatre or an environment capable of fulfilling guidelines for any other surgical operation.

• The person responsible for the operation must be available and capable of dealing with any complications which may arise.

• There should be close links with the patient's GP and community services for continuing care after the operation.

• Accurate records of all procedures and audit of results are essential.

American Urological Association 2018 [14] (Quality level 2+)
https://www.auanet.org/guidelines/guidelines/circumcision
Full statement:

The American Urological Association, Inc.® (AUA) believes that neonatal circumcision has potential medical benefits and advantages as well as disadvantages and risks. Neonatal circumcision is generally a safe procedure when performed by an experienced operator. There are immediate risks to circumcision such as bleeding, infection and penile injury, as well as complications recognized later that may include buried penis, meatal stenosis, skin bridges, chordee and poor cosmetic appearance. Some of these complications may require surgical correction. Nevertheless, when performed on healthy newborn infants as an elective procedure, the incidence of serious complications is extremely low. The minor complications are reported to be three percent.

Properly performed neonatal circumcision prevents phimosis, paraphimosis and balanoposthitis, and is associated with a markedly decreased incidence of cancer of the penis among U.S. males. In addition, there is a connection between the foreskin and urinary tract infections in the neonate. For the first three to six months of life, the incidence of urinary tract infections is at least ten times higher in uncircumcised than circumcised boys. Evidence associating neonatal circumcision with reduced incidence of sexually transmitted diseases is conflicting depending on the disease. While there is no effect on the rates of syphilis or gonorrhea, studies performed in African nations provide convincing evidence that circumcision reduces, by 50-60 percent, the risk of transmitting the Human Immunodeficiency Virus (HIV) to HIV negative men through sexual contact with HIV positive females. There are also reports that circumcision may reduce the risk of Human Papilloma Virus (HPV) infection. While the results of studies in other cultures may not necessarily be extrapolated to men in the United States at risk for HIV infection, the AUA recommends that circumcision should be presented as an option for health benefits. Circumcision should not be offered as the only strategy for HIV and/or HPV risk reduction. Other methods of HIV and/or HPV risk reduction, including safe sexual practices, should be emphasized. Circumcision may be required in a small number of uncircumcised boys when phimosis, paraphimosis or recurrent balanoposthitis occur and may be requested for ethnic and cultural reasons after the newborn period. Circumcision in these children usually requires general anesthesia.

The risks and disadvantages of circumcision are encountered early whereas the advantages and benefits are prospective. When circumcision is being discussed with parents and informed consent obtained, medical benefits and risks, and ethnic, cultural, religious and individual preferences should be considered.

Board of Directors, May 2017 (Revised)
Board of Directors, October 2018 (Reaffirmed)

World Health Organization (WHO) 2007 [15] (Quality level 2+)

There is compelling evidence that male circumcision reduces the risk of heterosexually acquired HIV infection in men by approximately 60%. Three randomised controlled trials have shown that male circumcision provided by well-trained health professionals in properly equipped settings is safe. WHO/UNAIDS recommendations emphasise that male circumcision should be considered an efficacious intervention for HIV prevention in countries and regions with heterosexual epidemics, high HIV and low male circumcision prevalence. Male circumcision provides only partial protection, and therefore should be only one element of a comprehensive HIV prevention package which includes: the provision of HIV testing and counselling services; treatment for sexually transmitted infections; the promotion of safer sex practices; the provision of male and female condoms and promotion of their correct and consistent use.

WHO is leading UN Agencies (UNAIDS, UNICEF and UNFPA) to set norms and standards, develop policy and programme guidance for safe male circumcision services and support countries to develop male circumcision policies and strategies within the context of a comprehensive HIV prevention strategy.

Royal Dutch Medical Association (Koninklijke Nederlandsche Maatschappij tot bevordering der Geneeskunst) 2010 [16] (Level 3)

Non-Therapeutic Circumcision of Male Minors states that non-therapeutic circumcision of male minors is a violation of children's rights to autonomy and physical integrity. Contrary to popular belief, circumcision can cause complications – bleeding, infection, urethral stricture and panic attacks are particularly common. KNMG is therefore urging a strong policy of deterrence. KNMG is calling upon doctors to actively and insistently inform parents who are considering the procedure of the absence of medical benefits and the danger of complications. Insofar as there are medical benefits it is reasonable to put off circumcision until the age at which such a risk is relevant and the boy himself can decide about the intervention, or opt for any available alternatives.

Supplementary Material 2. Numbers of publications obtained from PubMed to Sep 21, 2021 using as keyword "circumcision" together with keywords shown.

Keyword	Number	Keyword	Number
Public health	4410	Balanoposthitis	55
Urinary tract infection	398	Lichen sclerosus/balanitis xerotica	146/105
Sexually transmitted infection/STI	2029/160	Penile cancer/penis cancer	571/534
Human papillomavirus/HPV	221/216	Prostate cancer	72
Herpes simplex virus/HSV-2	128/111	Sexual function	451
Syphilis	118	Sensitivity	277
Trichomonas/Trichomoniasis	32/39	Satisfaction	348
Chancroid	36	Pleasure	82
Mycoplasma	14	Complications/adverse events	2136/227
Human immunodeficiency virus/HIV	2090/2064	Cost effectiveness	185
Chlamydia	59	Timing	71
Bacterial vaginosis	42	Best time	37
Microbiome	25	Policy	658
Hygiene	420	Prevalence	2892
Phimosis	699	Ethics	603
Paraphimosis	53	Anesthesia	610
Balanitis	288	Technique	3875

Supplementary Material 3. Scottish Intercollegiate Guidelines Network (SIGN) grading system for recommendations in evidence-based guidelines which provides definitions for levels of evidence [1].

1++	High quality meta-analyses, systematic reviews of RCTs, or RCTs with a very low risk of bias
1+	Well conducted meta-analyses, systematic reviews of RCTs, or RCTs with a low risk of bias
1–	Meta-analyses, systematic reviews of RCTs, or RCTs with a high risk of bias
2++	High quality systematic reviews of case-control or cohort studies; or high quality case-control or cohort studies with a very low risk of confounding, bias, or chance and a high probability that the relationship is causal
2+	Well conducted case-control or cohort studies with a low risk of confounding, bias, or chance and a moderate probability that the relationship is causal
2–	Case -control or cohort studies with a high risk of confounding, bias, or chance and a significant risk that the relationship is not causal
3	Non-analytical studies, e.g., case reports, case series
4	Expert opinion

Supplementary Material 4

Glossary

Chordee: Curvature of the penis due to scar tissue or abnormality of the corpora cavernosa (the blood containing tissue that supports an erection).

Dorsal: The back or upper surface.

Epispadias: A rare malformation of the penis in which the urethra ends in an opening on the upper aspect (the dorsum) of the penis.

Glans: The sensitive tip or "head" of the penis.

HIV/AIDS: Human Immunodeficiency Virus/Acquired Immune Deficiency Syndrome.

Hypospadias: A relatively common abnormality of the penis that appears as an abnormal opening of the penis (meatus) on the underside of the penis rather than at the end.

ICU: Intensive Care Unit

Meatus: Opening or passage.

Paraphimosis: A condition in which the foreskin, once pulled back behind the glans penis, cannot be brought down to its original position.

Phimosis: Narrowing of the foreskin opening, leading to an inability to retract the foreskin over the glans penis.

Urethra: The membranous tube through the body of the penis, through which urine is discharged from the bladder.

Urethritis: Inflammation of the urethra caused by infection.

Ventral: The anterior or lower surface.

References

[1] Harbour R, Miller J. A new system for grading recommendations in evidence based guidelines. BMJ. 2001; 323: 334–336.

[2] American AP. American Academy of Pediatrics Task Force on Circumcision. Circumcision policy statement. Pediatrics. 2012; 130(3): 585-586.

[3] American AP. American Academy of Pediatrics Task Force on Circumcision. Male circumcision. Pediatrics. 2012; 130(3): e756-e785.

[4] Centers for Disease Control and Prevention. Information for providers counseling male patients and parents regarding male circumcision and the prevention of HIV infection, STIs, and other health outcomes. 2018. Available at: https://stacks.cdc.gov/view/cdc/58456.

[5] Centers for Disease Control and Prevention. Background, Methods, and synthesis of scientific information used to inform "information for providers to share with male patients and parents regarding male circumcision and the prevention of HIV infection, sexually transmitted infections, and other health outcomes". 2018. Available at: https://stacks.cdc.gov/view/cdc/58457.

[6] Centers for Disease Control and Prevention. Peer Review Comments and CDC Responses for Information for Providers Counseling Male Patients and Parents Regarding Male Circumcision and the Prevention of HIV infection, Sexually Transmitted Infections, and other Health Outcomes* and Background, Methods, and Synthesis of Scientific Information Used to Inform "Information for Providers to Share with Male Patients and Parents Regarding Male Circumcision and the Prevention of HIV Infection, Sexually Transmitted Infections, and other Health Outcomes"**. 2018. Available at: https://www.cdc.gov/hiv/pdf/risk/MC-HISA-Round-1-Peer-Review-Comments-and-Responses.pdf.

[7] Centers for Disease Control and Prevention. Summary of Public Comments and CDC Responses to Public Comments for Information for Providers Counseling Male patients and Parents Regarding Male Circumcision and the Prevention of HIV Infection, Sexually Transmitted Infections, and Other Health Outcomes. 2018. Available at: https://www.cdc.gov/hiv/pdf/risk/MC-HISA-Public-Comments-and-Responses.pdf.

[8] Sorokan ST, Finlay JC, Jeffries AL. Newborn male circumcision. Position Statement. Canadian Paediatric Society. Paediatr Child Health. 2015; 20: 311–315.

[9] Morris BJ, Klausner JD, Krieger JN, Willcox BJ, Crouse PD, Pollock N. Canadian Paediatrics Society position statement on newborn circumcision: a risk-benefit analysis revisited. The Canadian Journal of Urology. 2016; 23(5): 8492–8502.

[10] Morris BJ, Klausner JD, Krieger JN, Willcox BJ, Crouse PD, Pollock N. Reply by authors - re: Canadian Pediatrics Society position statement on newborn circumcision: A risk-benefit analysis revisited. The Canadian Journal of Urology. 2017; 24(1): 8687–8692.

[11] British Medical Association. Non-therapeutic male circumcision of children toolkit. 2019. Available at: https://www.bma.org.uk/advice/employment/ethics/children-and-young-people/non-therapeutic-male-circumcision-of-children-ethics-toolkit (Accessed: Augest 15, 2019).

[12] Morris BJ, Krieger JN. Non-therapeutic male circumcision. Paediatr Child Health. 2020; 30: 102–107.

[13] Royal College of Surgeons. Male Circumcision: Guidance for Healthcare Practitioners. Available at: https://www.rcseng.ac.uk/library-and-publications/rcs-publications/docs/male-circumcision/ (Accessed: October 28, 2021).

[14] American Urological Association. Circumcision. 2018. (revised May 2017; reaffirmed October 2018) Available at: https://www.auanet.org/guidelines/circumcision (Accessed: April 17, 2019).

[15] World Health Organization and UNAIDS. New data on male circumcision and HIV prevention: policy and programme implications. 2007. Available at: http://who.int/hiv/mediacentre/MCrecommendations_en.pdf.
[16] Royal Dutch Medical Association (KNMG). Non-therapeutic circumcision of male minors. Utrecht: Royal Dutch Medical Association (KNMG). 2010. Available at: http://knmg.artsennet.nl/Publicaties/KNMGpublicatie/Nontherapeutic-circumcision-of-male-minors-2010.htm.
